# Sarcopenia: Current Insights into Molecular Mechanisms, Diagnostics, and Emerging Interventional Approaches

**DOI:** 10.3390/ijms26146740

**Published:** 2025-07-14

**Authors:** Siying Tu, Xiaoyu Hao, Shan Xu, Xingyi Jin, Wang Liao, Hui Xia, Shaokang Wang, Guiju Sun

**Affiliations:** 1 Key Laboratory of Environmental Medicine and Engineering of Ministry of Education, Department of Nutrition and Food Hygiene, School of Public Health, Southeast University, Nanjing 210009, China; 230248566@seu.edu.cn (S.T.); haoxiaoyu1386@126.com (X.H.); 220234094@seu.edu.cn (S.X.); 230239087@seu.edu.cn (X.J.); wangliao@seu.edu.cn (W.L.); huixia@seu.edu.cn (H.X.); gjsun@seu.edu.cn (G.S.); 2 Clinical Medical Research Center for Plateau Gastroenterological Disease of Xizang Autonomous Region, School of Medicine, Xizang Minzu University, Xianyang 712082, China

**Keywords:** sarcopenia, aging, muscle protein metabolism, precision medicine

## Abstract

With global population aging, muscle atrophy and functional decline—hallmarks of sarcopenia—pose growing challenges to public health and significantly impact the quality of life in older adults. The goal of this review is to present a thorough examination of the most recent developments in the study of sarcopenia, including the development of its pathophysiological mechanisms, diagnostic techniques, and multimodal intervention strategies. Particular attention is given to the role of declining sex hormones, such as testosterone and estrogen, as key drivers of anabolic resistance and muscle loss during aging. The review also addresses the current opportunities and challenges in translating basic research into effective clinical applications. Key focus areas include protein metabolism, mitochondrial dysfunction, chronic inflammation, and neuromuscular junction degeneration. Finally, it outlines future directions for precision classification, early detection, and personalized treatment, aiming to support interdisciplinary collaboration and shift sarcopenia management from reactive care to proactive, targeted intervention.

## 1. Introduction

By 2050, the world’s population aged ≥ 65 is expected to rise from roughly 727 million in 2020 to over 1.5 billion, effectively doubling the demographic at highest risk for muscle decline. Sarcopenia is a complex syndrome that is closely related to ageing and is characterized by a progressive loss of skeletal muscle mass, strength, and function [1,2]. Community-based studies, employing various diagnostic cut-offs, report sarcopenia prevalence between 10% and 27% among older adults, with Oceania showing the highest and Europe the lowest rates under the European Working Group on Sarcopenia in Older People (EWGSOP) and EWGSOP2 criteria [3,4]. In Asia, the Asian Working Group on Sarcopenia (AWGS) and AWGS2 identify prevalence estimates of 11.5% in men and 16.7% in women aged 75–79 years, rising to nearly 48% in those over 80 in 2019 [5,6]. Low- and middle-income countries, while underrepresented in epidemiological surveys, are poised to bear a growing burden due to rapid demographic shifts and limited access to diagnostic modalities. The 2025 Global Leadership Initiative on Sarcopenia (GLIS) consensus redefines sarcopenia through three criteria, namely, loss of muscle mass, low strength, and impaired physical function, emphasizing its role as a driver of frailty, disability, and mortality [7,8]. This disease entity was officially included in ICD-10 (M62.84) in 2019, marking its paradigm shift from “complication of aging” to “interventional disease”.

This multi-system disease originates from complex molecular mechanisms, including mitochondrial dysfunction, chronic inflammation, and protein homeostasis disorders (such as mTOR/ubiquitin–proteasome imbalance). At its core lies a disruption of protein homeostasis, where reduced anabolic signaling via mTORC1 coincides with heightened proteolytic activity through the ubiquitin–proteasome and autophagy–lysosome pathways, creating a widening “molecular scissors gap” that favors muscle breakdown over synthesis [9]. With the current management strategies following the “exercise–nutrition–drug” trinity model, resistance exercise training (RET) remains the cornerstone of treatment. Nutritional optimization, including leucine and vitamin D supplementation, can synergize with exercise by enhancing protein synthesis and reducing inflammation. Meanwhile, stem cell-based therapies, especially mesenchymal stromal cells (MSCs), have entered clinical trials for muscular dystrophy. Despite the progress, translating mechanistic insights into clinical practice remains challenging. Daily protein intake ≥ 1.5 g/kg can reverse mTORC1 resistance [10]. Vitamin D supplementation improves muscle function by inhibiting IL-6/STAT3 signaling [11]. Targeted drugs like myostatin inhibitor bimagrumab increased lean body mass by 5.2% in a phase II trial, but failed to improve exercise capacity, suggesting the need for combined intervention [12]; senolytics (dasatinib + quercetin) clear aging SCs, and preclinical models show 35% muscle strength recovery [13].

**Figure 1:**
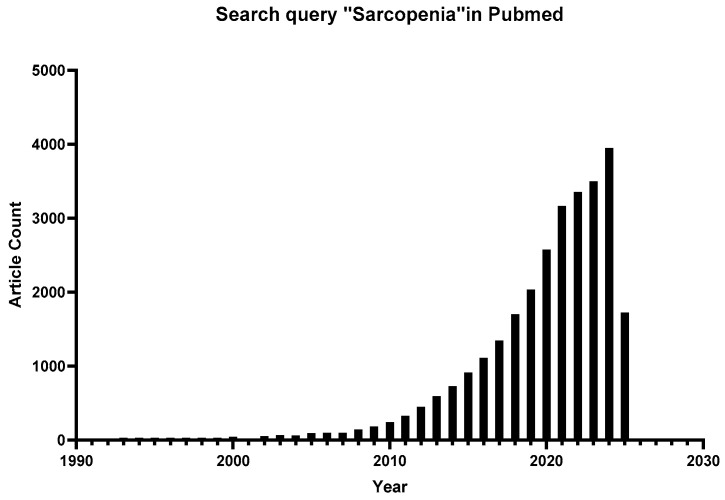
Number of published articles related to “sarcopenia” by year.

Sarcopenia has garnered increasing attention in recent years, as illustrated in Figure 1. Nevertheless, despite substantial advances in understanding its underlying mechanisms, significant gaps remain in translating these discoveries into clinical practice. Current research remains fragmented across disciplines, with limited studies integrating multisystem interactions (e.g., gut-muscle axis or immune-muscle crosstalk) into a holistic therapeutic framework. For example, while studies have highlighted the role of mitochondrial dysfunction and inflammatory aging [14,15,16,17], their synergistic effects on muscle atrophy have not been fully investigated. Different criteria exacerbate diagnostic challenges; for example, differences in the definition of “low grip strength” between EWGSOP2 and AWGS2019 have led to biased estimates of prevalence in Asian populations [6,18]. This review aims to use sarcopenia as a model for integrated geriatric care. Understanding these complexities is essential for developing scalable strategies to address its growing prevalence. As life expectancy continues to rise, redefining sarcopenia from a multisystem perspective is essential for shifting it from an irreversible marker of frailty to a modifiable target for healthy longevity. Sarcopenia is expected to transform from an “inevitable fate of old age” to a “preventable and reversible metabolic disease,” reshaping the future landscape of healthy aging. The current review paper extensively explains the pathophysiology and signaling mechanisms associated with sarcopenia, as well as recent therapies and therapeutic options. Furthermore, this study will give researchers and clinicians knowledge-based information to assist them in developing novel medicines and implementing modern interventional approaches.

## 2. Molecular Mechanisms: Decoding the Multisystem Interplay

**Figure 2:**
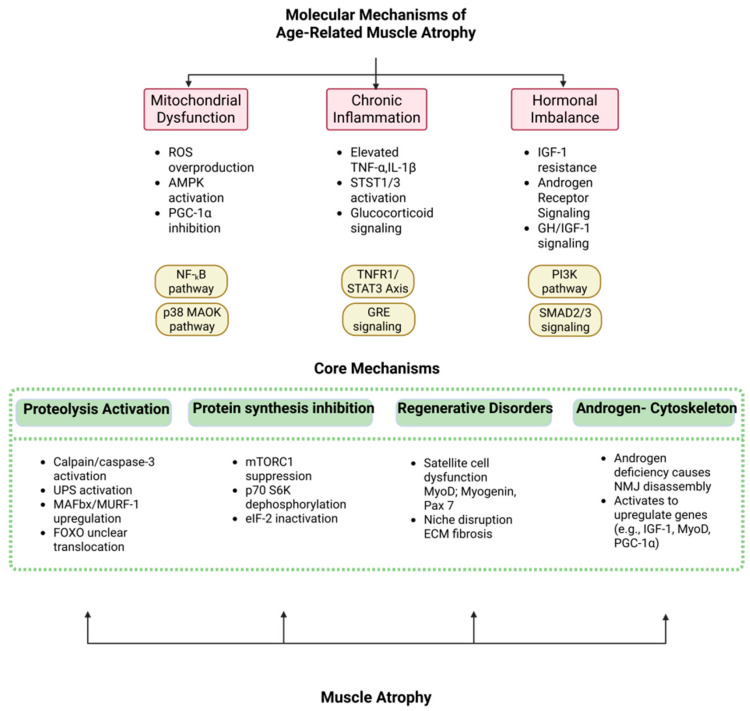
Core mechanisms of sarcopenia are driven by upstream molecular triggers.

The core mechanisms of sarcopenia lie in protein homeostasis imbalance: the “molecular scissors gap” between anabolic decline (mTORC1 signal resistance) and catabolic hyperactivity (UPS/autophagy overactivation) as appeared in Figure 2. Mitochondrial dysfunction is particularly prominent in aging muscle—PGC-1α expression decreases by 40–60%, leading to loss of oxidative phosphorylation capacity and accumulation of reactive oxygen species (ROS), triggering lipid peroxidation and mtDNA damage [15,16]. At the same time, chronic low-grade inflammation inhibits satellite cell (SC) regeneration through the IL-6/STAT3 pathway, while TNF-α-induced NF-κB activation upregulates E3 ubiquitin ligases (Atrogin-1, MuRF1), accelerating myofibril degradation. Single-cell sequencing revealed that overexpression of FGF2 in the aged muscle microenvironment drives SCs out of the quiescent state, abnormal activation of JAK-STAT signaling leads to premature exhaustion, and TGF-β1 secreted by fibro-adipocyte progenitor cells promotes interstitial fibrosis, forming an irreversible “regeneration desert” [19,20,21,22,23,24]. In addition, neuromuscular junction (NMJ) degeneration and α motor neuron loss trigger muscle fiber denervation and atrophy through the Wallerian degeneration pathway, forming a vicious cycle of “exercise–metabolism”.

### 2.1. Upstream Triggers

#### 2.1.1. Mitochondrial Disorders and Oxidative Injury

Skeletal muscle cells are highly dependent on oxidative metabolism, and their mitochondria are central in energy supply and metabolic regulation. However, with age and pathology, mitochondrial mass gradually declines, leading to impaired mitochondrial biosynthesis (e.g., PGC-1α-mediated processes) and diminished autophagy scavenging, resulting in a gradual accumulation of dysfunctional mitochondria in the cell [25,26]. This process is accompanied by the overproduction of ROS (e.g., superoxide, H_2_O_2_), which not only directly destroys the cellular structure, but also accelerates protein degradation through the activation of the expression of E3 ubiquitin ligases (e.g., Atrogin-1 and MuRF1) by activation of NF-κB and FoxO transcription factors, which leads to an imbalance in protein synthesis and degradation in skeletal muscle. In parallel, increased mitochondrial ROS have been associated with telomere damage and the senescence-associated secretory phenotype (SASP), accelerating cellular aging processes through telomere erosion and genome instability [20,27,28]. Studies have shown that cells acquiring a senescent phenotype commonly have high levels of mitochondrial ROS and single-strand breaks in the telomere region, which may further accelerate telomere erosion and trigger cellular senescence [29,30,31]. Although oxidative stress alone is not sufficient to cause muscle atrophy, relevant studies have pointed out that muscle mass is significantly affected only when ROS act synergistically with other dysregulatory factors such as mitochondrial dysfunction and inflammatory mediators [15,16]. Therefore, maintaining redox balance becomes a potential intervention strategy to delay the onset of sarcopenia.

#### 2.1.2. Chronic Low-Grade Inflammation

**Figure 3:**
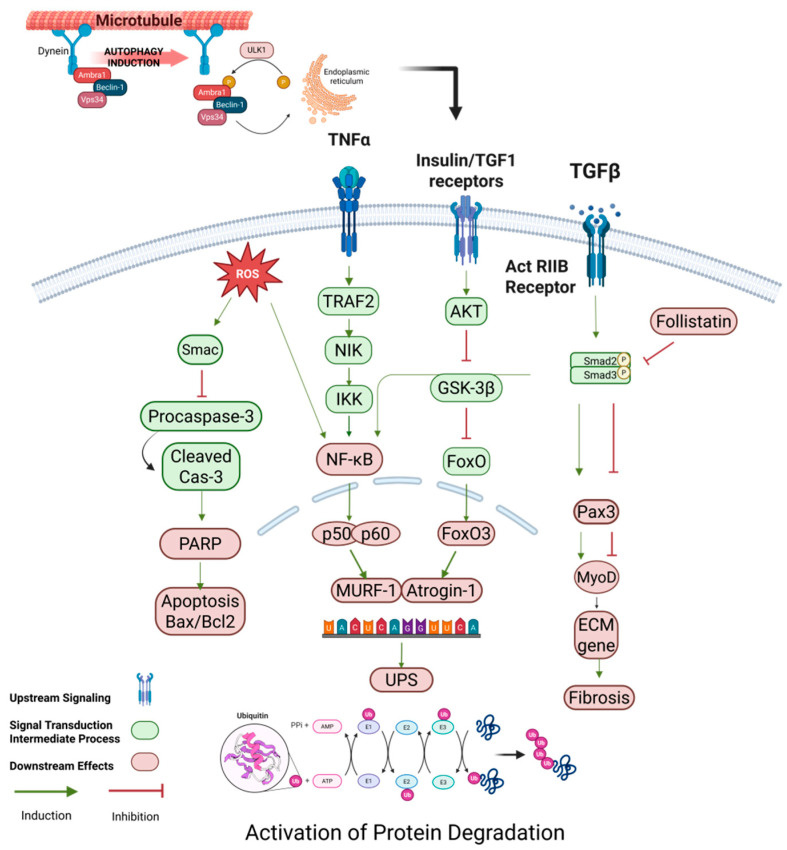
**Regulation of protein degradation in skeletal muscle.** This diagram illustrates the activation of protein degradation mechanisms in skeletal muscle, focusing on inflammatory and stress signaling pathways. TNFα, TGF-β, and ROS trigger the activation of the NF-κB pathway through TRAF2, NIK, and IKK, leading to the expression of MuRF1 and Atrogin-1, two muscle-specific E3 ligases involved in protein ubiquitination. TGF-β signaling also induces Smad2/3, which further promotes the activation of follistatin and inhibits myogenesis. On the other hand, ROS and NF-κB pathways enhance the UPS (ubiquitin–proteasome system) to drive protein degradation. Additionally, AKT and FoxO3 participate in cross-talk between anabolic and catabolic signals, where FoxO3 upregulates key catabolic genes like Atrogin-1. This network is central to muscle mass homeostasis and is often dysregulated in conditions like sarcopenia, where excessive protein breakdown contributes to muscle wasting. (Color scheme: Blue denote transmembrane receptors; green indicates intracellular signaling proteins; red lines indicate inhibition; green arrows indicate activation; orange circles denote phosphorylation events; and red/pink elements represent downstream translation machinery.) **Abbreviations:** TNFα: Tumor Necrosis Factor Alpha; ROS: reactive oxygen species; TRAF2: TNF receptor-associated factor 2; NIK: NF-κB-inducing kinase; IKK: IκB kinase; NF-κB: nuclear factor kappa-light-chain-enhancer of activated B cells; MuRF1: Muscle Ring Finger 1; Atrogin-1: Atrophy-Related Ubiquitin Ligase 1; Smad2/3: Mothers Against Decapentaplegic Proteins 2 and 3; AKT: protein kinase B; GSK-3β: glycogen synthase kinase 3 Beta; FoxO3: Forkhead Box O3; Pax3: Paired box 3; MyoD: myogenic differentiation 1; UPS: ubiquitin–proteasome system. Created using BioRender https://BioRender.com.

Under conditions of chronic disease, aging, and metabolic abnormalities, the body is often in a state of persistent low-grade inflammation. Pro-inflammatory factors such as TNF-α, IL-6, and CRP, by activating the TNFR1/STAT3 signaling pathway on the cell surface, not only directly inhibit Akt/mTOR signaling, thereby impairing protein synthesis, but also activate the UPS and autophagy systems simultaneously, accelerating protein degradation as shown in Figure 3 [32,33]. Numerous clinical studies have demonstrated a strong correlation between increased levels of inflammatory markers and the loss of muscle mass and strength in the elderly. For instance, patients with chronic obstructive pulmonary disease and cancer cachexia frequently exhibit a positive correlation between IL-6 levels and muscle loss. In addition, inflammation can further exacerbate disturbances in skeletal muscle protein metabolism indirectly by inhibiting the normal function of the growth hormone (GH) and insulin-like growth factor-1 (IGF-1) axis [34]. Moreover, inflammatory cytokines exacerbate insulin resistance, reducing nutrient uptake and further impairing protein metabolism in skeletal muscle [35,36]. While acute inflammation is necessary for muscle repair and regeneration, prolonged and sustained inflammation promotes protein degradation, inhibits regeneration, induces anabolic resistance and insulin resistance, and accelerates the onset and progression of sarcopenia. Therefore, targeting chronic inflammation represents a critical therapeutic avenue in the prevention and management of age-related muscle loss.

#### 2.1.3. Hormone and Growth Factor Imbalances

A number of hormones and growth factors closely control the ratio of muscle protein synthesis to breakdown, with insulin-like growth factor 1 (IGF-1) being a key player in increasing satellite cell proliferation and differentiation as well as protein synthesis. IGF-1, which is mostly produced in the liver, functions as a systemic growth factor by binding to its receptor and activating the PI3K/Akt signaling pathway. This leads to the activation of mTORC1, which in turn triggers the phosphorylation of 4E-BP and 70 kDa ribosomal protein S6 kinase, enhancing the efficiency of protein translation [37,38]. However, with aging and pathological stimuli, the levels of growth hormone, IGF-1, and mechano-growth factor (MGF) generally decrease, leading to a decrease in mTORC1 activity, and consequently, a decrease in the efficiency of protein synthesis [39]. In addition, a negative regulatory signal “ myoinhibitor of muscle growth” is upregulated at the level of a variety of myasthenic conditions, which activates the Smad2/3 pathway by binding to the ActRIIA/B receptor and inhibits myogenic regulators such as MyoD and Myogenin expression, further hindering muscle cell proliferation and differentiation [40,41,42]. It has been shown that overexpression of IGF-1 isoforms or inhibition of muscle growth inhibitor in transgenic animals partially restored muscle mass in aging, indicating the important role of IGF-1 in maintaining muscle morphology and function. Notably, aging also leads to decreased insulin sensitivity of myocytes, which may be related to increased deposition of ectopic lipids (e.g., ceramides), impaired mitochondrial function, and decreased endothelial function in the muscle, and all of these alterations directly or indirectly affect normal IGF-1 and insulin signaling, which exacerbates the loss of muscle strength and mass. Notably, androgen signaling via the androgen receptor (AR) is also crucial for muscle health, as aging is associated with a decline in circulating testosterone and diminished AR expression or sensitivity in skeletal muscle, contributing to anabolic resistance and reduced muscle mass and strength. Furthermore, AR signaling influences neuromuscular junction integrity and mitochondrial function, thereby affecting muscle quality beyond simple hypertrophy [30,43,44].

Taken together, three major upstream triggers, mitochondria disorder; chronic low-grade inflammation and hormone growth factor imbalances are collectively contributed to disturbances in skeletal muscle protein metabolism and sarcopenia. Understanding these mechanisms is important for developing targeted intervention strategies, for example, by improving mitochondrial function, decreasing inflammation levels, and restoring growth factor homeostasis, which could provide new ideas and targets for delaying or combating sarcopenia.

### 2.2. Activation of Protein Degradation Pathways

Imbalance in core protein metabolism plays a pivotal role in muscle atrophy and is mediated by the coordinated activation of four major proteolytic systems: UPS, the autophagy–lysosome system, calpains, and caspases. Among these, the UPS is the primary pathway for selective intracellular protein degradation, crucial for removing misfolded, damaged, or short-lived regulatory proteins. In skeletal muscle, the ubiquitin ligase (E3) ubiquitin ligases Atrogin-1 (also known as MAFbx) and MuRF1 are upregulated in catabolic states and specifically target key structural proteins such as myosin heavy chain and actin for 26S proteasome-mediated degradation. The process of UPS degradation occurs in two major steps: firstly, the UPS is activated by ubiquitin activating enzyme (E1), ubiquitin conjugating enzyme (E2) and E3 to form a polyubiquitin chain tag, which is followed by the recognition and unfolding of the substrate in an ATP-dependent manner by the 19S regulator of the UPS, which is ultimately sent to the protein hydrolysis core for degradation [42,44,45,46]. Regulatorily, FoxO transcription factors enter the nucleus upon deacetylase (e.g., SIRT1) inactivation and bind directly to the promoters of Atrogin-1 and MuRF1, which in turn upregulate their expression; this process often occurs in the presence of elevated inflammatory factors, such as TNF or IL-1, and stress signals that cause mitochondrial damage and increased ROS, and is accompanied by the activation of the NF-κB and AMPK pathway activation. Experimental data showed that hyperubiquitination of MyoD, a substrate of Atrogin-1, inhibited myotube formation during dexamethasone-induced myotube atrophy, and knockdown of Atrogin-1 reversed this effect [45,47,48,49]. In addition, although MuRF1 mainly interacts with muscle structural proteins, it is equally important for regulating protein degradation and maintaining muscle protein homeostasis; a comparison of proteasome activities between wild-type and MuRF1 knockout mice showed that 20S and 26S protease activities remained almost stable under MuRF1-deficient conditions, further demonstrating the critical role of this factor [45,50,51].

However, the UPS alone cannot initiate the degradation of intact myofibrillar proteins because it lacks the ability to dissociate contractile proteins such as actin and myosin from the cytoskeleton. for example, in Atg7 gene-deficient muscles, due to autophagic insufficiency, the expression of components of the UPS such as MAFbx and MuRF1 is upregulated, leading to a compensatory increase in protein degradation, which, in turn, results in a significant reduction in myofiber size [48]. This essential disassembly step is carried out by calpains and caspase-3. Calpains (especially μ-calpain/calpain-1 and m-calpain/calpain-2) are calcium-activated cysteine proteases that cleave Z-disk-associated proteins such as desmin and titin, thereby disrupting sarcomeric integrity and exposing substrates for UPS targeting. Caspase-3, traditionally known for its role in apoptosis, also contributes to muscle proteolysis by cleaving the actomyosin complex and generating a characteristic 14 kDa actin fragment. Furthermore, caspase-3 enhances proteasomal activity by cleaving regulatory subunits of the 26S proteasome. Notably, its transcription is directly stimulated by activated Stat3, forming a pathological feed-forward loop in conditions such as cancer cachexia, where blocking Stat3 phosphorylation can mitigate muscle loss [52,53,54,55].

In parallel, the autophagy–lysosome system is indispensable for the clearance of large protein aggregates, long-lived proteins, and dysfunctional organelles like mitochondria. During autophagy, cytoplasmic components are encapsulated by double-membraned autophagosomes, which subsequently fuse with lysosomes to form autolysosomes where degradation occurs. This process is regulated by autophagy-initiating proteins such as Unc-51 Like Autophagy Activating Kinase 1 (ULK1) and Beclin-1, and membrane-associated microtubule-associated protein light chain (3LC3), whose lipidated form (LC3-II) serves as a marker of autophagic flux. Autophagy is tightly controlled by nutrient and energy signals: mTORC1 inhibits autophagy under nutrient-rich conditions by phosphorylating ULK1, whereas AMPK activates autophagy during energy stress by enhancing ULK1 and Beclin-1 activity. FoxO3 also promotes autophagy by upregulating genes such as LC3 and Bnip3, thereby linking UPS and autophagy at the transcriptional level [25,37,41]. Clinically, increased LC3-II/LC3-I ratios, a marker of autophagy, have been observed in muscle biopsies from sarcopenic and cachectic patients, indicating heightened autophagic activity [52].

These four proteolytic systems do not function in isolation; rather, they exhibit intricate functional complementarity and compensatory interactions. For instance, in skeletal muscles deficient in Atg7—a key autophagy gene—there is compensatory upregulation of UPS components like Atrogin-1 and MuRF1, which paradoxically accelerates muscle protein degradation and leads to fiber atrophy [56]. Similarly, under inflammatory and oxidative stress conditions, parallel activation of calpain and caspase-3 is essential to facilitate myofibrillar breakdown and amplify the degradative process initiated by the UPS. Thus, while the UPS is central in targeted protein clearance, calpain and caspase-3 are indispensable for the initial disassembly of the cytoskeleton, and autophagy ensures the removal of damaged organelles and aggregates that the UPS cannot process. Together, these systems orchestrate a highly regulated but ultimately pathological breakdown of muscle tissue under chronic catabolic stimuli.

Overall, protein degradation systems—including the UPS, autophagy–lysosome pathway, and calcineurin/caspase-related pathways—are integral to the maintenance of muscle mass. These systems are finely regulated to recognize and remove damaged or no longer required proteins, preventing them from accumulating in cells as harmful aggregates. However, excessive degradation activity, due to a combination of stress, inflammation, hormonal imbalance, or aging, can upset the balance between protein synthesis and degradation, leading to muscle atrophy and loss of function. Meanwhile, intracellular deubiquitinating enzymes (e.g., USP19) are also involved in the regulation of protein homeostasis, and their expression levels positively correlate with key components of the UPS, and their knockdown significantly protects muscle mass in some models of myasthenia gravis, details are shown in Figure 3. In conclusion, these highly regulated degradation pathways not only maintain protein homeostasis in physiological states, but their aberrant activation in pathological states, especially under various catabolic conditions, is believed to be an important molecular basis for muscle atrophy and sarcopenia.

### 2.3. Protein Synthesis Inhibition

Protein synthesis inhibition has a critical role in the development of muscle atrophy, and its regulatory mechanism involves the coordinated dysregulation of multiple signaling pathways. The mTORC1 pathway plays a decisive role in promoting protein synthesis during normal growth and exercise. Mammalian target of rapamycin (mTOR) is a serine/threonine kinase that can be activated in response to a variety of internal and external signals such as nutrients, energy status, and growth factors. In muscle cells, upstream PI3K activation enables the activation of Akt, which subsequently regulates protein synthesis and degradation through multiple downstream pathways [44,50,57,58,59]. Specifically, Akt can phosphorylate and inactivate tuberous sclerosis complex-2, which activates mTORC1 and promotes the phosphorylation of the 70 kDa ribosomal protein S6 kinase (p70S6K) [36,60]; at the same time, by modulating the activities of the eukaryotic initiation factor 4E-binding protein (4E-BP) and the elongation initiation factors (eIF-2, eEF-2), mTORC1 enhances the translation initiation and elongation processes, ultimately increasing protein synthesis [41]. Conversely, insulin-independent pathways operate through leucine binding to Sestrin2, which relieves GATOR2 inhibition to load RagA/B GTPases for mTORC1 lysosomal recruitment [49]. In addition, Akt effectively reduces protein degradation by inhibiting the activity of glycogen synthase kinase-3β (GSK-3β) (activating eIF-2B) and negatively regulating the FOXO-mediated proteasomal pathway, thus realizing the dual regulation of protein synthesis and degradation. However, with aging or under conditions such as wasting muscle damage and low-protein diets, nutritional and hormonal signaling sensing is reduced, and activation of mTORC1 by amino acids (e.g., leucine) and insulin is markedly weakened, leading to blockage of ribosome biosynthesis (e.g., rRNA transcription) and translation initiation. Phosphorylation levels of p70S6K have been reported to decrease by ~50% in muscle of older adults compared to youth, suggesting that the mTORC1 pathway is functionally impaired during aging, further limiting muscle protein synthesis [26,37,50,52].

Meanwhile, opposing mTORC1-driven anabolism, the transforming growth factor-β (TGF-β)/myostatin axis exerts potent inhibition of muscle growth. Myostatin (GDF-8) forms a complex by binding to the ActRIIA/B receptor on the cell membrane, preferentially to the activin IIB type I receptor, which then induces phosphorylation of receptor-associated Smad proteins (mainly Smad2/3). The phosphorylated Smad2/3 forms a heterodimer with Smad4, and then transfers to the nucleus, initiating the transcription of a series of downstream target genes, which often inhibit the expression of myogenic differentiation factors, such as MyoD and Myogenin, as well as the expression of Pax3, thus limiting the proliferation of muscle cells [40,54,55]. Not only does muscle growth inhibitor play a negative regulatory role in developmental myogenesis, its level is upregulated in several myasthenia gravis and upregulates the expression of E3 ubiquitin ligases (e.g., MuRF1 and MAFbx) by decreasing Akt phosphorylation and activating the FOXO transcription factor, which further stimulates protein degradation [45,57]. In contrast, it has been shown that myoinhibitor gene deletion leads to a significant increase in Akt activity in ex vivo skeletal muscle, and that treatment of mice with monoclonal antibodies that block myoinhibitor (e.g., REGN1033) is effective in increasing muscle mass and strength and preventing further loss of muscle mass in models of myasthenia gravis that include braking and dexamethasone treatment [40].

In overall regulation, protein synthesis in skeletal muscle is also dependent on IGF-1 and insulin signaling. Binding of IGF-1 to its transmembrane tyrosine kinase receptor activates the PI3K/Akt pathway through phosphorylation of the receptor substrate, IRS-1, which both promotes protein synthesis and inhibits FOXO-mediated protein degradation, thereby enhancing muscle growth and regeneration on multiple levels. Insulin, another important anabolic hormone, is also involved in regulation through a similar pathway. However, myocyte sensitivity to insulin is significantly reduced during aging, which may be related to the deposition of ectopic lipids (e.g., ceramides) in muscle, impaired mitochondrial function, and decreased endothelial function. In addition, aging is often accompanied by attenuated PPARγ coactivator 1-α (PGC-1α) signaling, which not only further reduces Akt and mTOR expression, but also results in severely impaired muscle cell protein synthesis, thereby affecting overall muscle mass.

**Figure 4:**
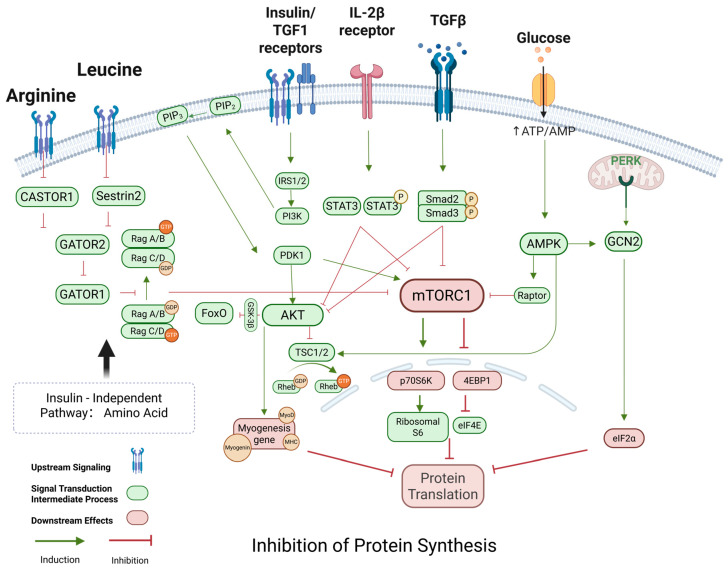
**Regulation of protein synthesis in skeletal muscle.** This figure illustrates the integration of anabolic and catabolic signals regulating muscle protein synthesis through mechanistic target of rapamycin complex 1 (mTORC1). Amino acids such as leucine and arginine activate mTORC1 via Rag GTPases and their upstream regulators (Sestrin2, CASTOR1, and GATOR complexes). Insulin/IGF1 signaling stimulates the PI3K-AKT pathway, which inhibits the TSC1/2 complex, thereby activating Rheb and mTORC1. Insulin/IGF1 and TGF-β pathways activate PI3K-AKT signaling, which suppresses the TSC1/2 complex to activate Rheb and mTORC1, promoting translation via p70S6K and 4EBP1/eIF4E. Conversely, catabolic signals such as AMPK, GCN2, PERK, TGF-β/Smad2/3, and IL-2β/STAT3 inhibit mTORC1 activity, leading to reduced protein synthesis. These regulatory mechanisms are central to muscle mass maintenance and are frequently dysregulated in sarcopenia. Abbreviations: mTORC1: mechanistic target of rapamycin complex 1; PI3K: Phosphoinositide 3-Kinase; IRS1/2: Insulin Receptor Substrate 1/2; AKT: protein kinase B; PDK1: 3-Phosphoinositide-Dependent Protein Kinase-1; TSC1/2: tuberous sclerosis complex 1/2; Rheb: Ras Homolog Enriched in Brain; p70S6K: ribosomal protein S6 kinase beta-1; 4EBP1: Eukaryotic Translation Initiation Factor 4E-Binding Protein 1; eIF4E: Eukaryotic Translation Initiation Factor 4E; eIF2α: Eukaryotic Translation Initiation Factor 2 Alpha; AMPK: AMP-activated protein kinase; GCN2: General Control Non-Depressible 2; PERK: PKR-Like Endoplasmic Reticulum Kinase; FoxO: Forkhead Box Protein O; MyoG: Myogenin; MHC: myosin heavy chain; IL-2β receptor: Interleukin-2 Beta Receptor; STAT3: Signal Transducer and Activator of Transcription 3; TGFβ: transforming growth factor beta; Smad2/3: SMAD Family Member 2/3. Sestrin2: Leucine Sensor Protein 2; CASTOR1: Cytosolic Arginine Sensor for mTORC1 Subunit 1; GATOR1/2: GTPase-Activating Protein Toward Rags Complexes. Created using BioRender https://BioRender.com.

To sum up the inhibition of protein synthesis is mainly stems from two major mechanisms: first is the impaired function of the mTORC1 pathway, which is regulated by nutritional, hormonal, and energy status, leading to insufficient key translation initiation factors and downstream signaling; second is the negative regulatory effect brought about by the activation of the TGF-β/Myostatin signaling pathway, which downregulates the muscle differentiation factors through Smad2/3 signaling and inhibits cell cycle progression and further exacerbate muscle atrophy through activation of protein degradation pathways. Meanwhile, IGF-1 and insulin signaling positively regulate protein synthesis, but aging and metabolic abnormalities reduce the activation of these pathways’ details are shown in Figure 4. It is the synergistic dysregulation of multiple signaling pathways that leads to the severe inhibition of protein synthesis in skeletal muscle in both physiological and pathological states, which provides the molecular basis for the onset and development of muscle atrophy.

### 2.4. Stem Cells and Regenerative Disorders

**Figure 5:**
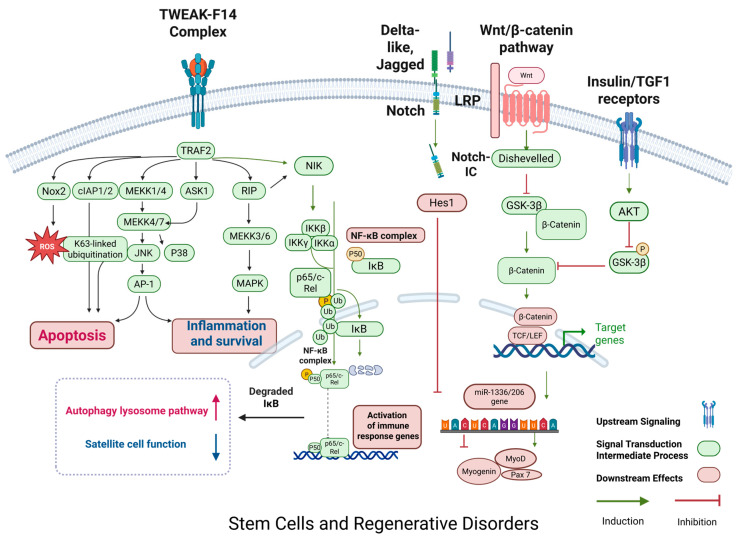
**Regulation of protein regenerative disorder in skeletal muscle.** This figure illustrates the activation of protein degradation mechanisms in skeletal muscle, focusing on inflammatory and stress signaling pathways. TNFα, TGF-β, and ROS trigger the activation of the NF-κB pathway through TRAF2, NIK, and IKK, leading to the expression of MuRF1 and Atrogin-1, two muscle-specific E3 ligases involved in protein ubiquitination. TGF-β signaling also induces Smad2/3, which further promotes the activation of follistatin and inhibits myogenesis. On the other hand, ROS and NF-κB pathways enhance the UPS (ubiquitin–proteasome system) to drive protein degradation. Additionally, AKT and FoxO3 participate in cross-talk between anabolic and catabolic signals, where FoxO3 upregulates key catabolic genes like Atrogin-1. This network is central to muscle mass homeostasis and is often dysregulated in conditions like sarcopenia, where excessive protein breakdown contributes to muscle wasting. (Color scheme: Blue and pink denote transmembrane receptors; green indicates intracellular signaling proteins; red lines indicate inhibition; green arrows indicate activation; orange circles denote phosphorylation events; and red/pink elements represent downstream translation machinery.) **Abbreviations:** Fn14: Fibroblast growth factor-inducible 14; TWEAK: TNF-like weak inducer of apoptosis; TRAF2: TNF receptor-associated factor 2; MEKK: mitogen-activated protein kinase; ASK1: Apoptosis signal-regulating kinase 1; RIP: receptor-interacting protein; JNK: c-Jun N-terminal kinase; P38: p38 mitogen-activated protein kinase; MAPK: mitogen-activated protein kinase; AP-1: activator protein 1; Nox2: NADPH oxidase 2; ROS: reactive oxygen species; cIAP1/2: cellular inhibitor of apoptosis protein 1/2; NF-κB: nuclear factor kappa-light-chain-enhancer of activated B cells; IKK: IκB kinase; IκB: inhibitor of kappa B; NIK: NF-κB-inducing kinase; Wnt: wingless-related integration site; TCF/LEF: T-cell factor/lymphoid enhancer-binding factor; GSK-3β: glycogen synthase kinase 3 beta; IGF1: insulin-like growth factor 1; AKT: protein kinase B; miR-133/206: MicroRNA-133 and MicroRNA-206; Myogenin: myogenic factor; MyOD: myogenic differentiation 1; Pax7: Paired box protein Pax-7; Notch-IC: Notch intracellular domain; Hes1: Hairy and enhancer of split-1. Created using BioRender https://BioRender.com.

The ability of satellite cells to differentiate progressively declines with age, and this decline stems from both intrinsic senescence drivers—such as Pax7 epigenetic silencing and mitochondrial stress/DNA damage within satellite cells—and extrinsic microenvironment alterations, including a TGF-β-enriched fibrotic niche and chronic inflammation or cytokine dysregulation. These combined factors collectively impair stem cell activation and differentiation [58]. This functional decline of satellite cells is not only reflected in the decrease in the number of differentiated cells but is also accompanied by a significant attenuation in the expression of myogenic markers such as Myf-5, MyoD, myogenin, and MRF4/Myf6 [39,59]. Reductions at both mRNA and protein levels reflect transcriptional dysregulation and impaired translational efficiency, thereby disrupting myogenic programming in aging muscle [59]. Which in turn leads to a reduced rate of differentiation of satellite cells to myotubes and deficient expression of myosin in the muscles of aged rats, thereby affecting the normal muscle fiber regeneration and repair. The molecular mechanism mainly involves an imbalance in the activity of the Notch and Wnt signaling pathways: under normal conditions, Notch signaling is rapidly activated after muscle injury to promote proliferation and self-renewal of satellite cells, whereas Wnt signaling tends to drive cells toward differentiation [60,61]. However, Notch signaling activity is significantly reduced during aging, while Wnt signaling tends to be hyperactive [35]. In youth, spatial segregation maintains a balance whereby Notch signaling predominates in satellite cells, while transient Wnt/β-catenin activation occurs only during injury to drive differentiation; this spatiotemporal control is mediated by niche-derived paracrine cues and disrupted in aging by chronic Wnt hyperactivity [62]. This imbalance not only directly inhibits the normal differentiation of satellite cells, but also leads to a decrease in intracellular expression of key genes, such as Pax7, and accelerates the depletion of the satellite cell reserve pool. In addition, inflammatory factors such as TNF-α also inhibit the Wnt/β-catenin pathway by upregulating the secreted antagonist Dickkopf-1 (Dkk1), which blocks LRP5/6 co-receptors, while simultaneously activating GSK-3β to promote β-catenin phosphorylation and proteasomal degradation [63,64,65]. Meanwhile, TWEAK, a member of the TNF-κB superfamily, activates NF-κB signaling by binding to the receptor Fn14 under tissue injury and inflammation, and its effects are concentration-dependent: at low concentrations (10–100 ng/mL), TWEAK tends to activate the bypass pathway (NIK/IKK), which promotes myofibril fusion and myofibril formation; whereas at higher concentrations of TWEAK (~500 ng/mL), it inhibits cell differentiation via the classical IKKβ pathway, which in turn induces muscle atrophy under pathological conditions [66,67]. This concentration-dependent duality of TWEAK in skeletal muscle shows how signal duration and strength dictate divergent cellular fates: low-dose TWEAK transiently promotes myoblast fusion, while sustained high doses trigger catabolic responses through persistent NF-κB/Nox2-ROS signaling, driving muscle atrophy [68,69]. In addition, TWEAK/Fn14 signaling is also capable of activating NADPH oxidase (Nox2)-dependent reactive oxygen species (ROS) generation, producing chronic ROS over hours to days, distinct from acute ROS bursts seen in exercise. Chronic ROS oxidize ryanodine receptors, causing calcium leakage and activating muscle atrophy programs. The TWEAK/Fn14 axis thereby induces sustained Nox2-derived ROS production, leading to persistent activation of catabolic pathways rather than transient redox signaling [70]. And the increase in ROS induced by chronic inflammation is closely associated with Nox2 expression and NF-κB activation, which collectively up-regulate the expression of E3 ubiquitin ligases such as MuRF1 and atrogin-1, and ultimately lead to the enhancement of proteasomal activities and the muscle protein degradation. Specifically, MuRF1 ubiquitinates myofibrillar proteins such as myosin heavy chain, desmin, and troponin to promote thick filament degradation, while atrogin-1 targets translation initiation factors like eIF3-f, inhibiting protein synthesis. Together, these processes drive muscle atrophy [57,71,72,73]. On the other hand, remodeling of the extracellular matrix (ECM) also plays an indispensable role in this process: the ability of TGF-β1 to strongly stimulate collagen deposition leads to the gradual formation of fibrosis, and this accumulation of fibrosis significantly impedes the regeneration of damaged muscle fibers [25,43,74,75,76]. TGF-β1-driven fibrosis is partially reversible in early stages through interventions such as senolytics or pirfenidone, which mitigate ECM accumulation; however, persistent ECM stiffening mechanically traps satellite cells in a quiescent state, impairing their motility and activation, and thereby hampering muscle regeneration. Satellite cells not only rely on their intrinsic molecular mechanisms for proliferation and differentiation, while studies of heterochronic parabiosis demonstrate that rejuvenating the aged niche restores satellite cell function, confirming the dominance of extrinsic regulation over intrinsic cellular deficits. Recent study from 2023 demonstrates that senescent cells are key negative regulators within the skeletal muscle regenerative niche, the authors define a conserved senescence signature and show that targeting senescent cells SASP or their secretome CD36 can enhance muscle regeneration in both young and aged mice [77]. But their normal function is also dependent on a microenvironment (niche) composed of multiple cellular components, biophysical and biochemical factors [78,79]. This niche provides a stable platform for satellite cells to maintain their quiescent state until activation by containing molecules such as proteoglycans. For example, heparan sulfate proteoglycans (HSPGs) and small leucine-rich proteoglycans (SLRP) serve as dynamic reservoirs for growth factors such as FGF2, HGF, and TGF-β. HSPGs sequester these factors via sulfated heparan sulfate chains. Upon tissue injury or inflammation, heparanase cleavage of heparan sulfate chains or matrix metalloproteinase (MMP)-mediated degradation of SLRP core proteins triggers controlled release of these growth factors, thereby activating regenerative or fibrotic programs [80,81,82]; at the same time, the synergistic action of IGF-1 and TGF-β and other pathway members has a significant effect on the regulation of the activity of satellite cells [73,81]. In recent years, muscle growth inhibitor and its antagonist, the secretory glycoprotein follicle inhibitor, have also attracted much attention. Among them, muscle growth inhibitor, as an important negative regulator of the TGF-β superfamily, inhibits the proliferation and differentiation of satellite cells and reduces skeletal muscle mass, whereas follicle inhibitor, by antagonizing the action of muscle growth inhibitor overexpression of follicular inhibin in transgenic mice was shown to significantly increase muscle mass, whereas deletion of this factor resulted in abnormal skeletal structure and impaired muscle development [64,83]. In addition, the complex interplay between Notch and the Wnt/β-catenin signaling pathway in the regulation of muscle regeneration has been widely discussed, and although classical Wnt/β-catenin signaling antagonizes Notch signaling under some conditions to promote myogenic differentiation, it has also been reported that its activation in some cases induces instead the proliferation and self-renewal of satellite cells, which in turn inhibiting myogenic differentiation [35,60,61,81,84,85,86,87]. This dynamic antagonism and context-dependent synergy critically regulate muscle regeneration, sustained Wnt activation promotes differentiation by suppressing Notch, while transient Wnt activation (e.g., post-injury) or high ligand concentration—especially with PI3K co-signaling—induces satellite cell proliferation and self-renewal, delaying differentiation [88]. At the same time, aging leads to a decrease in the overall activity of satellite cells, which negatively affects muscle regeneration by slowing down the proliferation rate, reducing the response to proliferative stimuli, and shortening telomeres; even in the absence of significant damage, aging satellite cells continue to secrete excessive amounts of FGF2, which disrupts the cellular quiescent state, and further weakens their self-renewal ability details are shown in Figure 5. The regenerative repair capacity of skeletal muscle is further diminished by the reduction in the number of motor neurons, the redistribution of muscle fiber types, and changes in the external environment that accompany ageing [89].

### 2.5. Androgen Receptor Signaling and Cytoskeletal Stability

**Figure 6:**
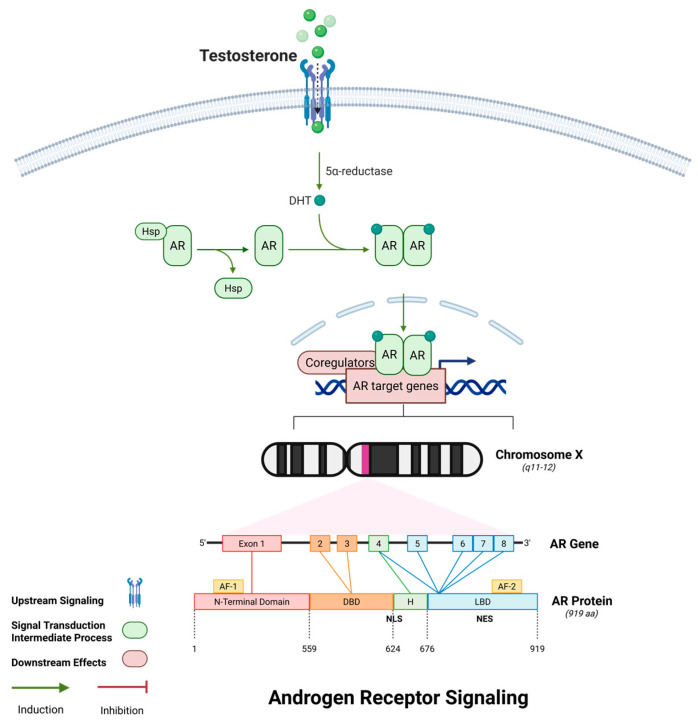
AR signaling in skeletal muscle. This figure illustrates the molecular signaling cascade initiated by testosterone. Upon entering the cell, testosterone is converted to its more potent form dihydrotestosterone (DHT) via 5α-reductase. DHT binds to cytoplasmic androgen receptors (AR), releasing them from heat shock proteins (Hsp). The AR-DHT complex undergoes conformational change, dimerizes, and translocates into the nucleus, where it binds androgen response elements (AREs) in the DNA to regulate transcription of AR target genes, including those related to muscle growth and differentiation. The lower panel shows the genomic organization of the AR gene on the X chromosome (Xq11–12), and the domain structure of the AR protein, including the N-terminal domain (NTD), DNA-binding domain (DBD), hinge region with nuclear localization signal (NLS), and ligand-binding domain (LBD). (Color scheme: Purple denotes transmembrane receptors; green indicates intracellular signaling proteins; red lines indicate inhibition; green arrows indicate activation; orange circles denote phosphorylation events; and red/pink elements represent downstream translation machinery.) Abbreviations: AR: androgen receptor; DHT: dihydrotestosterone; Hsp: heat shock protein; ARE: androgen response element; NTD: N-terminal domain; DBD: DNA-binding domain; LBD: ligand-binding domain; NLS: nuclear localization signal; NES: nuclear export signal. Created using BioRender https://BioRender.com.

AR is a pivotal regulator of skeletal muscle homeostasis throughout life, acting through both genomic and non-genomic mechanisms. With aging, circulating androgen levels—particularly testosterone—decline significantly, contributing to muscle atrophy, impaired regeneration, and anabolic resistance, which are hallmark features of sarcopenia. Structurally, AR comprises four functional domains: an N-terminal transactivation domain (NTD), a central DNA-binding domain (DBD), a hinge region containing the nuclear localization signal (NLS), and a ligand-binding domain (LBD). Upon ligand binding—typically by dihydrotestosterone (DHT)—AR undergoes conformational changes, dissociates from chaperones such as HSP90, dimerizes, and translocates into the nucleus, as shown in Figure 6 [90]. There, it binds androgen response elements (AREs) and recruits co-regulators to modulate the transcription of target genes.

At the genomic level, AR signaling promotes muscle growth and metabolic support by upregulating anabolic genes including IGF-1, MyoD, Myogenin, PGC-1α, and SOD2. These genes coordinate satellite cell activation, mitochondrial biogenesis, oxidative metabolism, and antioxidant defense, collectively enhancing protein synthesis and muscle regeneration [91]. AR also potentiates PI3K/Akt/mTOR signaling and represses FoxO-mediated expression of atrophy-related genes such as Atrogin-1 and MuRF1, counteracting proteasomal degradation. In aged muscle, the downregulation of AR expression and function contributes to blunted anabolic responses to nutrition and exercise—a phenomenon known as anabolic resistance [92,93,94,95]. Beyond transcriptional regulation, AR also exerts rapid, non-genomic effects through interactions with cytoskeletal and membrane-associated proteins. A well-characterized example is its complex formation with Filamin A, a cytoskeletal scaffold that crosslinks actin filaments and mediates mechanical signal transduction. AR–Filamin A complexes localize at the sarcolemma, focal adhesion sites, and NMJs, where they stabilize cell-matrix connections, support mechanotransduction, and maintain cytoskeletal tension during contraction [96]. Aging disrupts this complex, leading to actin disorganization, sarcolemmal fragility, and NMJ fragmentation—early structural defects preceding overt muscle loss. In vitro studies using C2C12 myoblasts show that androgen-induced AR/FlnA signaling protects cells from oxidative stress-induced senescence, whereas its disruption triggers mitochondrial dysfunction and increased expression of senescence markers [97]. These findings broaden the understanding of AR/FlnA interactions as potential therapeutic targets for skeletal muscle diseases. Another study published in 2025 investigates early gene expression changes in skeletal muscle of pre-symptomatic SBMA mice, focusing on transcriptional alterations at the neuromuscular junction and muscle regions. It identifies loss of key NMJ and sarcomere genes, dysregulated calcium flux, and demonstrates that pharmacological activation of SERCA with CDN1163 partially rescues muscle function and size, highlighting calcium modulation as a potential therapeutic strategy [98]. In the neuromuscular context, AR is essential for NMJ maintenance. It regulates acetylcholine receptor (AChR) clustering, postsynaptic membrane organization, and motor neuron connectivity. Androgen deficiency, as seen in AR knockout or castrated animal models, leads to reduced AChR density, NMJ disassembly, and impaired synaptic transmission—features that mimic age-associated neuromuscular degeneration [42]. Notably, testosterone replacement therapy (TRT) and selective androgen receptor modulators (SARMs) have been shown to restore NMJ structure and function, improve grip strength, and enhance motor coordination in aged animals [99].

Moreover, AR signaling intersects with inflammatory and oxidative pathways, repressing pro-inflammatory cytokines such as IL-6 and TNF-α and preserving a regenerative microenvironment. Post-translational modifications—including phosphorylation and ubiquitination—further fine-tune AR activity under stress conditions. Together, these multifaceted functions position AR as not only an anabolic regulator but also a structural stabilizer and neuromuscular protector. As such, therapeutic restoration of AR signaling—whether through hormonal replacement, SARMs, or downstream mimetics—holds promise for mitigating sarcopenia and age-related functional decline.

## 3. Diagnostic Advancements: From Traditional to Precision Tools

Over the past decade, five major international consortia have developed distinct yet overlapping frameworks for the diagnosis of sarcopenia: EWGSOP/EWGSOP2, AWGS/AWGS2, IWGS, FNIH, and SDOC. In 2010, EWGSOP first formalized a three-step algorithm requiring low muscle mass, diminished strength, and impaired physical performance, assessed via DXA or BIA, hand-grip dynamometry, and gait speed testing, respectively. A comprehensive update (EWGSOP2) published in 2018 elevated low grip strength (<27 kg men; <16 kg women) as the principal entry criterion and recommended confirmation with quantitative muscle-mass measures and poor performance (gait ≤ 0.8 m/s) to stage severity [2,3,5,6]. Building on EWGSOP’s approach, AWGS issued region-specific cut-offs in 2014 that reflected anthropometric and lifestyle differences in Asian populations. AWGS2 (2019) introduced the category “possible sarcopenia” (low muscle strength with or without slow gait) to prompt early community-based screening, while full diagnosis still hinges on low appendicular lean mass plus objective strength or performance deficits [100,101]. The International Working Group on Sarcopenia (IWGS) likewise emphasizes muscle mass and function but places less emphasis on strength alone. Its 2011 consensus defines sarcopenia by low muscle mass combined with poor mobility—particularly chair-rise ability and gait speed—highlighting patients at high risk of functional decline [102]. In contrast, the Foundation for the National Institutes of Health (FNIH) Sarcopenia Project advocates a body-mass-index–adjusted lean-mass index (ALM/BMI) rather than height-adjusted ASM, and recommends a hierarchical assessment beginning with gait speed, followed by grip strength, and finally muscle-mass quantification via DXA. This adjustment aims to improve specificity by accounting for adiposity [103]. Most recently, the Sarcopenia Definitions and Outcomes Consortium (SDOC) simplified diagnosis by requiring only muscle weakness and slowness, omitting lean-mass criteria owing to its weaker predictive power for adverse outcomes. SDOC’s higher grip-strength thresholds (e.g., <35.5 kg men) and gait speed cut-offs aim to maximize case-finding sensitivity for falls and disability [104]. The details of definitions and criteria of sarcopenia are described in Table 1.

All five frameworks agree that muscle-strength and performance metrics are central, yet they diverge on whether and how to incorporate quantitative mass measures. EWGSOP, AWGS, IWGS, and FNIH include low muscle mass as an essential criterion, whereas SDOC relies solely on functional markers. The inclusion of sarcopenia in ICD-10-CM (M62.84) in 2016 underscores its recognition as a treatable condition [6]. To harmonize global practice and improve diagnostic validity, further research must clarify whether a three-domain model (mass, strength, function) or a two-domain model (strength and function) best predicts clinically meaningful outcomes across diverse populations.

### 3.1. Traditional Tools

#### 3.1.1. Screening Questionnaires

The Strength, Assistance in Walking, Rising from a Chair, Climbing Stairs, and Falls (SARC-F) questionnaire is one of the most widely used tools for sarcopenia screening. Developed by Malmström and Morley in 2013 and recommended by EWGSOP2, it offers the advantages of rapid self-assessment, simplicity, and high specificity (63–91%), though it exhibits only moderate sensitivity (27–77%). Notwithstanding being initially designed as a diagnostic tool, SARC-F is now primarily used for screening, particularly in clinical and community settings [106,107]. It assesses five self-reported domains: muscle strength, need for assistance in walking, ability to rise from a chair, ability to climb stairs, and history of falls. Higher scores are associated with decreased physical function and impairments in activities of daily living (ADL). Its ease of use and independence from physician interpretation make it highly practical. However, due to its relatively low sensitivity, several modified versions have been proposed, including the SARC-CalF, which adds calf circumference (CC) to improve diagnostic accuracy [108]. The SARC-CalF shows improved sensitivity (57.4–65.5%) compared to the original SARC-F (31.5–44.8%) and demonstrates higher overall diagnostic accuracy (75.7–79.2% vs. 64.3–70%). SARC-CalF consists of the same five items as SARC-F plus a sixth assessing CC. A total score ≥ 11 indicates a positive screening result, making it a more effective tool in primary care [109].

Another screening instrument, the Mini Sarcopenia Risk Assessment (MSRA), is available in two versions: a full seven-item (MSRA-7) and a shortened five-item version (MSRA-5) [110]. MSRA evaluates risk factors including age, hospitalization, physical activity, meal frequency, dairy and protein intake (MSRA-7 only), and unintentional weight loss. Cut-off scores of ≤30 for MSRA-7 and ≤45 for MSRA-5 suggest sarcopenia risk [111]. In a Chinese community population, MSRA-5 demonstrated superior diagnostic accuracy (85%) compared to MSRA-7 (70%), likely due to its simplicity and relevance to lifestyle factors. Compared with SARC-F, MSRA-5 showed higher sensitivity (90.2% vs. 29.5%) but lower specificity (70.6% vs. 98.1%). Although SARC-F maintains high specificity, MSRA-5 may be more effective for initial risk detection [112]. Comparison is shown in Table 2, and further validation in diverse populations is needed to optimize the clinical utility of these screening tools.

#### 3.1.2. Recommendations for Diagnosing

In clinical practice, handgrip strength is the most widely used surrogate for muscle strength, with the Jamar dynamometer regarded as the gold standard. According to EWGSOP2 criteria, grip strength < 27 kg in men and < 16 kg in women indicates muscle weakness [6]. Owing to its simplicity and practicality, both the Global Leadership Initiative on Malnutrition (GLIM) and EWGSOP incorporate grip strength as a key indicator for diagnosing malnutrition and assessing muscle mass. Notably, the updated EWGSOP2 guidelines emphasize its role not just in screening but also as a primary diagnostic tool.

For patients without access to a dynamometer, the five-repetition chair stand test is recommended. This involves rising from a chair without arm support five times in succession, with a completion time > 13 s indicating a risk of sarcopenia [113]. While the chair stand test does not directly assess muscle mass, it is considered a more holistic indicator of functional capacity and may detect decline earlier than grip strength alone. The 4 m gait speed test is another standardized and reliable method for evaluating physical performance. A walking speed ≤ 0.8 m/s is associated with severe functional limitations and is used by EWGSOP2 as a threshold for severe sarcopenia. The Short Physical Performance Battery (SPPB), comprising tests of static balance (parallel, semi-tandem, and tandem stance), 4 m gait speed, and the chair stand test, provides a comprehensive functional assessment. Scored from 0 to 12, a total score ≤ 8 indicates poor physical performance [114]. The SPPB is widely adopted in clinical and research settings and takes approximately 10–15 min to complete. Another practical assessment is the Timed Up and Go (TUG) test, which measures the time taken to rise from a chair, walk 10 feet, turn, return, and sit down. A time > 20 s is strongly predictive of poor physical function. The TUG test is validated and widely used across various countries and healthcare systems [115].

### 3.2. Precision Tool

#### 3.2.1. Dual-Energy X-Ray Absorptiometry (DXA) in Diagnosis of Sarcopenia

DXA is an established method for assessing body composition, capable of measuring three key components: lean mass (LM), fat mass (FM), and bone mineral density (BMD), with the added advantage of regional estimation. DXA is characterized by its rapid scan time, typically less than 20 min for a whole-body assessment and cost-effectiveness. However, a notable limitation is its inability to distinguish between muscle and intramuscular adipose tissue, thus limiting its capacity to directly assess true muscle mass [116]. Despite this limitation, DXA has been endorsed by major international sarcopenia working groups, including the EWGSOP, FNIH, IWGS, and AWGS [3,101,103]. A key concern raised by these groups is the feasibility of implementing DXA in all clinical settings, especially in low- and middle-income countries where access to advanced imaging may be limited [117]. According to the AWGS, DXA remains the most widely used imaging modality in sarcopenia research, whereas bioelectrical impedance analysis (BIA) is preferred for community screening due to its affordability, portability, ease of use, non-invasiveness, radiation-free nature, and rapid results. Importantly, both the EWGSOP and IWGS recognize that cut-off values for low muscle mass using DXA differ across populations; for example, the thresholds in Asian populations are generally lower than those in Caucasians [101,118,119]. This may partly explain the relatively lower prevalence of sarcopenia reported in Asian studies. Furthermore, cultural and lifestyle differences contribute to variability in outcomes, underscoring the need for population-specific diagnostic criteria. After considerable deliberation, experts concluded that DXA should be conditionally recommended for identifying low lean body mass in the diagnosis of sarcopenia. While DXA offers several advantages including the ability to estimate appendicular skeletal muscle mass (ASM) within minutes and low radiation exposure, its use remains largely confined to hospital settings and may be influenced by factors such as hydration status. Moreover, DXA provides only a quantitative assessment, without qualitative information about muscle tissue. Feedback from older adults involved in the external review of the ICFSR guidelines indicated a preference for clinical evaluation over costly or time-consuming imaging [120]. Similarly, health economics reviewers questioned whether the diagnostic benefit of DXA justified its expense in routine practice. Compared to CT and MRI, which are more commonly used in chronic disease management, DXA is simpler and more affordable, albeit less precise. Nonetheless, it is preferred over BIA in clinical research because it directly measures body composition, while BIA relies on population-specific prediction equations.

#### 3.2.2. Bioelectrical Impedance (BIA) in Diagnosis of Sarcopenia

BIA is a non-invasive, low-cost, rapid, and radiation-free method for assessing body composition and evaluating physical function. It is simple to administer and widely accessible. BIA operates on the principle of bioelectrical impedance, which involves applying a low-level alternating current (typically 50 kHz) through the body and measuring the opposition to the flow of this current. The impedance is composed of resistance and reactance, from which the phase angle—a biomarker reflecting cell membrane integrity and body cell mass—is derived. Tissues with higher water and electrolyte content conduct electricity better than fat or bone, enabling the estimation of body composition [121]. However, BIA has certain limitations, particularly in individuals with obesity or malignancies, where discrepancies between body weight and electrical conductivity can affect accuracy. Notably, BIA cannot directly measure appendicular skeletal muscle mass (ASM) or differentiate between intracellular and extracellular water compartments. Moreover, it does not directly quantify muscle fat content or muscle mass, but rather estimates these parameters based on the differential conduction of electrical currents through various tissues [122]. Despite these limitations, BIA has demonstrated utility in assessing body composition at specific time points and tracking longitudinal changes. Its portability, reproducibility, and applicability in both ambulatory and bedridden populations make it a versatile and cost-effective tool in clinical and community settings [123]. The revised EWGSOP2 guidelines recognize phase angle, derived from BIA, as a valuable indicator of muscle status. However, the EWGSOP2 consensus does not define specific cutoff thresholds for phase angle in diagnosing low muscle mass, though population-specific reference ranges have been reported. The accuracy of BIA-derived measurements is influenced by several factors, including age, ethnicity, hydration status, disease states, recent food intake, and physical activity [124]. Given the wide range of commercially available BIA devices, EWGSOP2 recommends relying on raw output values from each instrument, using the manufacturer-provided predictive equations. While not as precise as imaging modalities such as DXA, BIA has been endorsed by both European and Asian sarcopenia guidelines as a valid, portable alternative for muscle mass assessment, particularly when access to imaging is limited.

#### 3.2.3. Computed Tomography (CT)/Magnetic Resonance Imaging (MRI) as the Gold Standards

CT and MRI are considered gold-standard techniques for body composition assessment, particularly for evaluating lean body mass (LBM). These imaging modalities offer high-resolution visualization of body compartments and can accurately differentiate between fat and other soft tissues. However, both are associated with high costs and, in the case of CT, increased exposure to ionizing radiation compared to DXA [122]. CT imaging is currently the gold standard for quantifying muscle mass in patients with sarcopenia, cachexia, and frailty, CT images help to differentiate between various tissues and assess body composition based on the specific attenuation of each tissue measured in Hounsfield units (HU) [125]. Despite its widespread use in clinical and research settings, the absence of standardized protocols for image acquisition and analysis has been highlighted as a major limitation. A 2018 review by Engelke et al. noted considerable variability in CT imaging parameters across studies, hindering consistency and comparability [126]. The gold standard methods for assessing body composition are MRI and CT scanning. Both MRI and CT are capable of quantifying muscle mass and distinguishing it from adipose tissue, yet CT carries a significant drawback in terms of radiation exposure, which limits its feasibility for routine clinical screening [116]. Nevertheless, CT remains valuable for monitoring sarcopenia and related muscle-wasting conditions, especially in the context of chronic diseases such as cancer [116]. Despite their diagnostic strengths, MRI and CT share several limitations: (a) high operational costs, (b) lack of portability, (c) substantial space requirements, and (d) the need for highly trained personnel. Furthermore, standardized cutoff values for low muscle mass using these imaging methods have not yet been uniformly established, which poses a challenge for consistent clinical implementation.

#### 3.2.4. Ultrasound Imaging

Ultrasound imaging has emerged as a promising tool for evaluating skeletal muscle morphology, offering the ability to measure structural parameters such as muscle thickness, cross-sectional area, fascicle length, and pennation angle, as well as tissue echogenicity. A recent 2025 study reported that ultrasound demonstrated a sensitivity of 0.85 and a specificity of 0.74 in detecting muscle alterations [127]. Furthermore, its validity for quantifying muscle mass in older adults (≥60 years) has been reported with correlation coefficients ranging from 0.92 to 0.99 when compared to reference methods [128]. In practice, thigh muscle thickness (TMT) is often assessed using axial images obtained at the midpoint between the greater trochanter and the patella. While the rectus femoris is the most commonly evaluated muscle, other muscle groups such as the biceps brachii, triceps brachii, and gastrocnemius have also been utilized in thickness measurements. The growing interest in ultrasound for both clinical and research applications is attributed to its affordability, accessibility, portability, and lack of ionizing radiation. Ultrasound offers a convenient approach for serial assessment of muscle mass, enabling the detection of progressive muscle atrophy over time. Comparative studies have shown good validity of ultrasound-based assessments relative to established imaging modalities such as DXA, CT, and MRI. While the EWGSOP2 guidelines recognize ultrasound as a valid method for evaluating muscle mass, they currently do not provide specific cutoff thresholds for its application [6]. In contrast, the AWGS2 guidelines do not endorse ultrasound for muscle mass estimation, citing a lack of sufficient supporting evidence. Accordingly, further research is warranted to validate predictive equations for muscle mass estimation using ultrasound in both sarcopenic individuals and those with comorbid conditions.

CT and MRI are regarded as the gold standards for non-invasive assessment of skeletal muscle mass and quality due to their high sensitivity, specificity, and ability to quantify fat infiltration. However, their high cost, limited accessibility, and requirement for hospital-based equipment—along with radiation exposure in the case of CT—restrict routine use. In contrast, DXA offers a faster, lower-cost, and reproducible method for estimating regional lean mass with minimal radiation, though it lacks the ability to distinguish intramuscular fat from muscle tissue and may yield variable results depending on device manufacturer, patient age, and sex. BIA provides a portable, low-cost alternative with reasonable correlation to DXA, but its accuracy is compromised in older adults due to its dependence on hydration status and population-specific prediction equations. Emerging as a practical and radiation-free modality, ultrasound enables real-time evaluation of muscle morphology, including thickness, cross-sectional area, fascicle length, and echogenicity. Among these parameters shown in Table 3, echogenicity is particularly valuable, as it correlates with muscle quality and can indicate pathological changes such as myosteatosis or myofibrosis. Beyond these established modalities, several advanced technologies are gaining attention for their potential to assess neuromuscular and microstructural features of sarcopenia. High-density electromyography (HD-EMG) enables spatial mapping of motor unit potentials, offering sensitive detection of denervation and impaired motor unit recruitment [129]. Shear wave elastography (SWE) assesses mechanical stiffness of muscle tissue in real time, which may reflect fibrotic changes associated with chronic sarcopenia [130]. Near-infrared spectroscopy (NIRS) provides functional insights into muscle oxygenation and perfusion, although it does not capture anatomical or structural information [131]. Although these emerging methods are currently limited to research or specialized settings, they expand the diagnostic landscape by addressing aspects of muscle physiology not accessible through conventional imaging, and may serve as valuable adjuncts in early detection or phenotyping of sarcopenia.

### 3.3. Current Landscape of Sarcopenia Biomarkers

Sarcopenia is a multifactorial geriatric syndrome characterized by progressive muscle loss, involving intersecting molecular pathways reflected in diverse biomarker categories. Chronic low-grade inflammation is a hallmark of sarcopenia, with elevated levels of IL-6 (≥4.9 pg/mL) and TNF-α promoting proteolysis via STAT3 and NF-κB activation, respectively [132]. IL-1β, IL-18, and novel mediators like IL-27 and suPAR further disrupt myogenesis through inflammasome signaling [132]. These inflammatory markers are often elevated in sarcopenic individuals and correlate with reduced muscle mass, strength, and regenerative potential.

Oxidative stress and mitochondrial dysfunction contribute to muscle atrophy via reactive oxygen species (ROS) accumulation. Reduced activities of SOD, catalase, and GPx impair redox balance, while elevated urinary 8-OHdG (≥10 ng/mg creatinine) signals DNA damage [133]. Decline in Nrf2 and PGC-1α expression leads to mitochondrial biogenesis failure and reduced oxidative capacity. Mitochondrial-derived peptides such as humanin are under investigation as novel stress biomarkers [26]. Hormonal imbalances also play a pivotal role. IGF-1, a key activator of the PI3K/AKT/mTOR pathway, declines significantly with age. Testosterone and DHEA-S promote satellite cell activation and oppose myostatin expression, while estrogen and GH influence mitochondrial function and anabolic signaling [134]. FGF21 and melatonin, with metabolic and antioxidant properties, are emerging as potential biomarkers linking endocrine dysfunction to sarcopenia. NMJ degradation is increasingly recognized in sarcopenia pathogenesis [42]. Circulating levels of the C-terminal agrin fragment and neurofilament light chain reflect NMJ fragmentation and motor unit loss. BDNF and neuregulin-1 support NMJ repair and satellite cell function; their decline is associated with reduced exercise tolerance and motor deficits in aging populations. Muscle growth regulation is tightly controlled by the balance between negative modulators such as myostatin, activins, and GDF-15, and positive factors including follistatin, BMPs, BDNF, and irisin [42,86,96]. Elevated myostatin and activin A promote muscle wasting through Smad2/3 signaling, while downregulation of follistatin and myonectin impairs muscle regeneration. Clinical trials targeting this axis, such as the ActRIIB inhibitor bimagrumab, highlight the therapeutic relevance of this pathway. Muscle damage and regenerative biomarkers provide additional diagnostic value. While CK and LDH reflect acute muscle injury, they lack specificity in chronic sarcopenia. In contrast, the upregulation of E3 ligases Atrogin-1 and MuRF1 indicates sustained proteolysis [41,45]. Reduced nuclear expression of MyoD and myogenin signifies impaired regeneration. Epigenetic modulation of FGF2 and increased periostin expression have been linked to fibrosis and regeneration failure in aging muscle, as shown in Table 4.

In general, integrating inflammatory, hormonal, neuromuscular, mitochondrial, and regenerative biomarkers offers a comprehensive strategy for early diagnosis, phenotypic stratification, and therapeutic targeting of sarcopenia. Further research should validate novel candidates in large, diverse cohorts and translate biomarker insights into clinically actionable tools.

## 4. Interventions and Strategies for Skeletal Muscle Atrophy

Sarcopenia affects up to 29% of older adults in community healthcare settings. Management strategies include non-pharmacological and pharmacological approaches, with resistance training being the most effective non-drug intervention. Adequate nutritional intake—particularly protein, vitamin D, antioxidants, and long-chain polyunsaturated fatty acids—has shown positive effects on muscle preservation. Currently, no FDA-approved pharmacological treatments exist for sarcopenia, though several agents (e.g., growth hormone, anabolic steroids, SARMs, myostatin inhibitors) are under investigation with varying degrees of efficacy.

### 4.1. Nutritional and Exercise Interventions

Nutritional strategies remain a cornerstone in the prevention and management of sarcopenia. High-quality protein intake, particularly leucine-rich sources such as whey, plays a central role in stimulating muscle protein synthesis via activation of the mTORC1 pathway [1]. Compared to soy, whey protein enhances muscle anabolism by 30–40% more efficiently due to its rapid digestibility and higher leucine content. Plant-based proteins, while sustainable, require a higher intake (~60 g) to elicit a comparable anabolic response to 35 g of whey protein [135,136]. Additionally, supplementation with n-3 polyunsaturated fatty acids (PUFAs), especially EPA and DHA, has demonstrated anti-inflammatory and anti-catabolic effects. EPA suppresses TNF-α-induced apoptosis and enhances myotube survival by up to 25%, whereas DHA reduces the expression of the E3 ligase MuRF1, a key regulator of muscle degradation [52]. Antioxidants such as polyphenols (e.g., green tea extract) downregulate NF-κB activity, decrease serum creatine kinase levels, and mitigate exercise-induced muscle damage [137]. Moreover, vitamin D, often deficient in older adults (with 68% showing serum 25(OH)D levels < 20 ng/mL), supports muscle regeneration through Notch pathway activation and has been shown to improve post-injury muscle repair by 19% [138,139]. Importantly, triple supplementation with whey protein, vitamin D, and EPA enhanced muscle strength by 28% in a Phase III trial (NCT04512379). However, the challenge of anabolic resistance in aging muscles, along with nutrient safety thresholds (e.g., hypercalcemia risk at >4000 IU/day of vitamin D), must be carefully considered. Exercise, particularly resistance and endurance modalities, is fundamental to sarcopenia treatment. Resistance training activates anabolic signaling via the IGF-1/Akt/mTORC1 axis, suppresses catabolic processes including autophagy and ubiquitin–proteasome activation, and enhances satellite cell proliferation [140]. Endurance training stimulates mitochondrial biogenesis through PGC-1α activation, improves insulin sensitivity, and reduces systemic inflammation [141]. These adaptations collectively contribute to improved muscle quality, enhanced neuromuscular function, and delayed sarcopenic progression. Notably, eccentric resistance exercises are more effective in inducing sarcomeric expansion, whereas concentric modalities promote metabolic adaptations. Supplementation with specific amino acids such as leucine, HMB, arginine, and creatine monohydrate can further enhance muscle strength and hypertrophy, particularly when combined with resistance training. Creatine, for instance, increases intramuscular total creatine content by 25–37%, supporting ATP regeneration during exercise. ESPEN also recommends a minimum protein intake of 1.0 g/kg/day, adjusted for comorbidities and physical activity level [142]. Mao et al. provides significant insights into the protein needs of older adults in China. With the aging population expected to exceed 300 million by 2025, understanding adequate protein intake becomes increasingly crucial. By employing the Indicator Amino Acid Oxidation (IAAO) method to assess the protein requirements of healthy adults aged 65 and over, reveal that their estimated average requirement (EAR) and recommended nutrient intake (RNI) are 0.91 g/kg/d and 1.17 g/kg/d [143]. This study offers valuable data on the protein requirements of China’s elderly population, advocating for a reevaluation of dietary recommendations to address their specific nutritional needs, which is vital for future public health policies, and laid the foundation for 2023 The Chinese Nutrition Society’s new RDA of protein. Huang, in 2021, examined whether increasing protein intake to 1.3 g/kg/d improved visceral fat accumulation and serum cardiovascular risk markers more than the recommended daily allowance (RDA) in functionally limited older adults [144].

### 4.2. Pharmacological and Molecular Interventions

Pharmacological approaches to sarcopenia increasingly target molecular regulators of muscle mass. Myostatin, a TGF-β family member that negatively regulates muscle growth, is a key therapeutic target. Inhibition of myostatin with agents like LY2495655 led to significant improvements in gait and functional mobility in older adults [12], though results in genetic muscle disorders like Becker muscular dystrophy were less promising [145]. IGF-1, acting through the PI3K/Akt/mTOR pathway, suppresses catabolic mediators such as FoxO, Smad3, and NF-κB, making it a central anabolic factor. Other emerging targets include TNF-α blockers, SHIP2 inhibitors, GSK3β inhibitors, and mTOR activators [146,147,148]. Furthermore, the modulation of microRNAs (miRNAs), including miR-206 and miR-29, has shown potential to regulate myogenic differentiation and satellite cell function, although clinical translation remains in early stages [149]. These findings open new avenues for sarcopenia therapy, emphasizing the integration of molecular and lifestyle interventions shown in Table 5.

## 5. Challenges and Future Directions

Recent years have witnessed substantial progress in the understanding, diagnosis, and treatment of sarcopenia, an age-related loss of skeletal muscle mass and function. While sarcopenia has long been recognized as a hallmark of biological aging, its pathophysiological complexity and diagnostic ambiguity have delayed the development of standardized clinical approaches. However, with growing recognition of sarcopenia as a geriatric syndrome with significant implications for morbidity, disability, and healthcare burden, attention has shifted toward precision diagnostics, mechanism-driven therapies, and integrative treatment models.

A critical obstacle in sarcopenia management has been the lack of harmonized diagnostic criteria. While EWGSOP2 and AWGS provide regional cutoffs, variability in muscle mass estimation methods limits global applicability. In response, the ISarcoPRM algorithm was introduced in 2024 to integrate physical performance metrics with accessible imaging, namely quadriceps muscle ultrasound, enhancing diagnostic accuracy across diverse clinical settings [150]. Non-invasive modalities such as near-infrared spectroscopy (NIRS) and microwave-based muscle analysis are also emerging as portable and radiation-free tools capable of assessing muscle oxygenation, tissue quality, and intramuscular fat infiltration, offering promising adjuncts for early detection and longitudinal monitoring [151]. Furthermore, AI-powered platforms analyzing ultrasound-derived muscle texture have demonstrated superiority over DXA in predicting early declines in muscle quality, laying the groundwork for scalable, bedside sarcopenia screening [152].

Therapeutically, non-pharmacological interventions remain the first line of defense, with resistance training (RT) and tailored nutrition forming the foundation. A 2024 randomized controlled trial demonstrated that hybrid exercise—combining progressive resistance training with Tai Chi—significantly improved lower limb strength, balance, and appendicular skeletal muscle mass in older adults, highlighting the synergistic potential of multimodal physical therapies [153]. Nutritional strategies targeting anabolic resistance, such as leucine-enriched protein supplementation, vitamin D optimization, and omega-3 fatty acid intake, have shown incremental benefits, particularly when paired with structured exercise regimens [154,155]. On the pharmacological front, therapeutic development has accelerated, with several candidates entering late-stage trials. Among these, LPCN 1148—a novel oral androgen receptor agonist—has shown significant efficacy in increasing lean body mass and reducing hepatic encephalopathy recurrence in cirrhotic men with sarcopenia. The promising results from its Phase II trial earned it FDA Fast Track designation in 2024, signaling regulatory momentum in this area [156]. Similarly, TNF Pharmaceuticals in 2024 announced that MYMD-1 as a selective TNF-α inhibitor originally developed for autoimmune disorders has demonstrated biomarker improvement and functional gains in a Phase IIa trial, with a pivotal Phase IIb trial underway [157]. Although myostatin inhibitors like bimagrumab have previously increased muscle mass without functional gains, the focus has shifted toward combinatorial therapies targeting mitochondrial function, inflammation, and satellite cell activation.

Mechanistic insights have uncovered several therapeutic targets central to muscle degeneration. Senescent satellite cells and impaired mitochondrial biogenesis have emerged as key contributors to age-related myofiber atrophy. Preclinical studies show that the activation of M2 macrophages can rejuvenate satellite cell function and enhance myogenesis through the IL-4/STAT6 axis [158]. Likewise, metabolic interventions targeting the NAD^+^/SIRT1 pathway—such as nicotinamide riboside and microbiota-derived nicotinic acid—have demonstrated efficacy in restoring mitochondrial function and reducing oxidative stress in sarcopenic muscle [159,160,161]. In line with these findings, the ketogenic metabolite β-hydroxybutyrate (β-HB) has been shown to suppress protein breakdown and promote mitochondrial biogenesis via histone β-hydroxybutyrylation, reducing sarcopenic decline by over 30% in older individuals undergoing resistance exercise [162,163]. Emerging frontiers include gene and cell-based therapies, which promise transformative outcomes. AAV-Pax7 gene therapy has restored satellite cell function and increased myofiber cross-sectional area by 22% in aged mouse models, while CRISPR-Cas9-mediated myostatin knockout has achieved up to 25% muscle mass gain in dystrophic settings [164,165]. These breakthroughs underscore the potential of regenerative medicine in reversing muscle loss. Meanwhile, selective androgen receptor modulators (SARMs), engineered to preserve anabolic effects without the adverse profiles of testosterone, are currently in Phase II trials, showing improvements in strength and myofiber hypertrophy with minimal androgenic side effects [166].

Together, these innovations point toward an integrative, precision-based future for sarcopenia management. By combining advanced diagnostics, targeted pharmacology, and regenerative strategies, researchers are gradually redefining sarcopenia from an inevitable aging consequence to a modifiable condition with actionable interventions. Future progress demands integration of multi-omics data for precision diagnosis, investment in pathway-specific drug development, and global collaboration to address health disparities. By embracing these directions, the future of sarcopenia treatment will move towards more personalized, precision-based approaches, transforming sarcopenia into a modifiable aspect of aging.

## Figures and Tables

**Table 1:** International working group on diagnosis of sarcopenia.

International Working Group	Year	Recommendation for Diagnosing Sarcopenia	Notes
EWGSOP [2]	2010	Diagnosis requires both low muscle mass and either low muscle strength or low physical performance. Defines stages: pre-sarcopenia, sarcopenia, severe sarcopenia.	Introduced muscle function into definition. Muscle mass cut-offs: 2 SD below healthy young adults.
EWGSOP2 (Revision) [6]	2018	Low muscle strength is the primary screening criterion; confirmed by low muscle mass/quality. Poor physical performance defines severity.	Provided clear thresholds: Grip strength < 27 kg (men), <16 kg (women); gait speed ≤0.8 m/s. Prioritized strength as key prognostic factor.
AWGS [100]	2014	Diagnosis requires low muscle mass plus low muscle strength and/or low physical performance.	Regional thresholds for Asians: ASM (DXA) < 7.0 kg/m^2^ (men), <5.4 kg/m^2^ (women); grip strength < 26 kg (men), <18 kg (women); gait speed < 0.8 m/s.
AWGS2 (Revision) [101]	2019	Introduced “possible sarcopenia” (low strength or performance only). Full diagnosis: low muscle mass + low strength or performance.	Gait speed threshold raised to <1.0 m/s; emphasized early detection and community screening.
IWGS [102]	2011	Diagnosis: low muscle mass + poor physical performance (gait speed < 1.0 m/s). Muscle strength not required.	Recommended DXA to assess lean mass. Focused on older adults with mobility limitations.
FNIH Sarcopenia Project [103]	2014	Low grip strength (<26 kg men, <16 kg women) and low ALM/BMI (<0.789 men, <0.512 women) define sarcopenia.	Introduced ALM/BMI as a muscle mass index. Emphasized function-based approach.
SDOC [105]	2020	Sarcopenia defined as low muscle strength (grip: <35.5 kg men, <20 kg women) and slow gait speed (<0.8 m/s). Muscle mass not included.	Prioritized functional measures to predict outcomes like falls and disability.
SCWD [104]	2019	Classification into pre-sarcopenia (only low mass), sarcopenia (mass + strength/performance loss), and severe sarcopenia (all three impaired).	Proposed clinical staging framework. No specific numeric thresholds.
GLIS [8]	2024	Conceptual definition: includes muscle mass, muscle strength, and muscle-specific force; physical performance viewed as an outcome.	First global conceptual consensus; sets foundation for future harmonized operational criteria.

BIA = bioelectrical impedance analysis; DXA = dual-energy X-ray absorptiometry; LBM = lean body mass; ALM = appendicular lean mass; BMI = body mass index.

**Table 2:** Screening questionnaires comparison.

Tool	Items	Sensitivity	Specificity	Notes
SARC-F [106]	5 self-reported domains: strength, walking assistance, chair rise, stairs, falls	4–35%	80–98%	High specificity but poor sensitivity
SARC-Calf [109]	SARC-F + calf circumference	66.1%	73.8%	Adds calf circumference to boost both sensitivity and specificity
MSRA-5 [112]	Age, hospitalization, activity, meals, unintentional weight loss	59.7%	46.4%	More sensitive than SARC-F but lower specificity
MSRA-7 [112]	MSRA-5 items + dairy intake, protein intake	14.5%	88.7%	Highest specificity of MSRA versions, lowest sensitivity

**Table 3:** Screening tools comparison.

Modality	Principle	Diagnostic Performance	Advantages	Limitations	Notes	Cut-Off Points
BIA	Measures body impedance to estimate lean and fat mass based on conductor properties of tissues.	Sensitivity ~60%, specificity ≥ 85% (vs DXA)	Portable, rapid, low-cost, non-invasive; suitable for bedridden/mobile settings	Affected by hydration, electrode placement, equations; cannot measure intramuscular fat	Recognized by EWGSOP2 and AWGS as valid screening in resource-limited contexts	SMI < 7.0 kg/m^2^ (men), <5.7 kg/m^2^ (women) (EWGSOP2)
DXA	Low-dose X-rays differentiate bone, fat, and lean tissue to quantify appendicular lean mass.	Correlates strongly with CT/MRI; used as reference standard	Fast (<20 min), reproducible (same machine), regional analysis, low radiation	Cannot detect intramuscular adipose tissue; less accessible in low-resource settings; hydration effects	Endorsed by EWGSOP, AWGS, FNIH and IWGS; part of ICD-10 codes M62.84	ALM/height^2^ < 7.0 kg/m^2^ (men), <5.5 kg/m^2^ (women) (EWGSOP2); ALM/BMI < 0.789/0.512 (FNIH)
CT	X-ray attenuation differentiates tissues by Hounsfield units, quantifying muscle cross-sectional area and fat infiltration.	High accuracy; excellent agreement with MRI	Precise quantification of muscle and fat; gold standard for muscle-quality assessment	High radiation dose; costly; limited portability; no standardized global cut-offs	Used mainly in research and oncology follow-up	SMI < 55 cm^2^/m^2^ (men), <39 cm^2^/m^2^ (women)
MRI	Magnetic fields and radiofrequency pulses produce high-contrast images of muscle volume and fat infiltration.	Comparable to CT; no ionizing radiation	Non-ionizing, multiplanar, excellent soft-tissue contrast; assesses composition and architecture	High cost; time-consuming; limited access; space and technologist requirements	Interchangeable with CT for muscle mass assessment	SMI < 55 cm^2^/m^2^ (men), <39 cm^2^/m^2^ (women)
Ultrasound Imaging	High-frequency sound waves measure muscle thickness, cross-sectional area, and echogenicity.	ICC 0.92–0.99 for thickness measures; correlates with DXA/CT	Portable; radiation-free; low-cost; real-time; assesses muscle quality via echogenicity	Operator-dependent; lack of standardized protocols and cut-offs; limited penetration depth	Growing interest for bedside and community use; needs further validation	Rectus femoris thickness < 1.0–1.5 cm (varies; no universal cut-off)

SMI = skeletal muscle index; ALM = appendicular lean mass.

**Table 4:** Key biomarker categories in sarcopenia.

Category	Biomarker	Function/Mechanism
Inflammatory	IL-6, TNF-α, CRP, IL-1β, IL-18, suPAR	Promote catabolism via STAT3, NF-κB; inhibit regeneration
Oxidative Stress/Mitochondria	SOD, GPx, Catalase, 8-OHdG, Nrf2, PGC-1α, Humanin	Indicate mitochondrial dysfunction and redox imbalance
NMJ and Motor Units	CAF, NFL, BDNF, Neuregulin-1	Reflect NMJ degradation and axonal injury
Hormonal	IGF-1, Testosterone, GH, Estrogen, DHEA-S, FGF21, Melatonin	Regulate protein synthesis and satellite cells via PI3K/Akt/mTOR
Muscle Growth Regulators	Myostatin, Activin A/B, GDF-15, follistatin, BMP-7, BDNF, Irisin, Myonectin	Balance anabolic and catabolic signals
Muscle Damage and Turnover	CK, LDH, Atrogin-1, MuRF1, MyoD, Myogenin, FGF2 methylation, Periostin	Reflect muscle atrophy and impaired regeneration

**Table 5:** Nutritional intervention strategies for skeletal muscle atrophy.

Intervention	Mechanism of Action	Key Outcomes
Whey Protein	Activates mTORC1 via leucine-rich amino acid profile	Increase muscle protein synthesis (~30–40% vs. soy) will lead an increase of strength
Plant Protein	Provides essential AAs but lower leucine bioavailability	Requires higher intake (~60 g) to match whey effect
n-3 PUFAs (EPA/DHA)	Decrease TNF-α signaling, MuRF1 inhibition	Increase Myotube survival (25%) will decrease muscle catabolism
Polyphenols	Inhibit NF-κB activation	Decrease Inflammation will decrease creatine kinase and protection against muscle damage
Vitamin D	Modulates Notch signaling, enhances regeneration	Increase Post-injury recovery (19%) lead to regenerative decline, but risk of hypercalcemia at high doses
Triple Supplement	Combined anabolic, anti-inflammatory, and regenerative effects	Increase muscle strength by 28% (Phase III trial)
Resistance Training	Activates IGF-1/Akt/mTOR, stimulates satellite cells	Increase muscle mass and strength will decrease catabolism and increase Type II fiber activation
